# Recent Advances on Synthetic and Polysaccharide Adhesives for Biological Hemostatic Applications

**DOI:** 10.3389/fbioe.2020.00926

**Published:** 2020-08-14

**Authors:** Dawei Li, Jing Chen, Xing Wang, Mingming Zhang, Chunlin Li, Jin Zhou

**Affiliations:** ^1^Eighth Medical Center of the General Hospital of the Chinese People’s Liberation Army, Beijing, China; ^2^Department of Orthopedics, Aerospace Center Hospital, Beijing, China; ^3^Beijing National Laboratory for Molecular Sciences, Institute of Chemistry, Chinese Academy of Sciences, Beijing, China; ^4^University of Chinese Academy of Sciences, Beijing, China; ^5^The People’s Liberation Army Strategic Support Force Characteristic Medical Center, Beijing, China

**Keywords:** hemostatic, hydrogel, adhesives, polysaccharides, tissue regeneration

## Abstract

Rapid hemostasis and formation of stable blood clots are very important to prevent massive blood loss from the excessive bleeding for living body, but their own clotting process cannot be completed in time for effective hemostasis without the help of hemostatic materials. In general, traditionally suturing and stapling techniques for wound closure are prone to cause the additional damages to the tissues, activated inflammatory responses, short usage periods and inevitable second operations in clinical applications. Especially for the large wounds that require the urgent closure of fluids or gases, these conventional closure methods are far from enough. To address these problems, various tissue adhesives, sealants and hemostatic materials are placed great expectation. In this review, we focused on the development of two main categories of tissue adhesive materials: synthetic polymeric adhesives and naturally derived polysaccharide adhesives. Research of the high performance of hemostatic adhesives with strong adhesion, better biocompatibility, easy usability and cheap price is highly demanded for both scientists and clinicians, and this review is also intended to provide a comprehensive summarization and inspiration for pursuit of more advanced hemostatic adhesives for biological fields.

## Introduction

Traumatic closure, wound repair and regeneration of surgical damaged soft tissues often occur medically. Uncontrolled bleeding, as a main cause of trauma-related deaths, frequently occurs on the battlefield and in the operating room. It is reported that 50% of military deaths stem from the bleeding (Behrens et al., 2014). In general, coagulation is a complicated process of plasma transformation from an unstable platelet plug to stable and insoluble fibrin. At the initial stage, formation of initial platelet plug can bind to the injured endothelium to achieve the stable adhesion and trigger the aggregation of locally activated platelets to form a hemostatic plug and stop the bleeding. Then, coagulation cascade, including intrinsic and extrinsic coagulation pathways with start-up modes and contributory coagulation factors, is activated immediately to accomplish hemostasis. Although body’s own coagulation process played important roles in preventing the excessive bleeding from the death, formation of stable blood clots or insoluble fibrin including primary hemostasis and clotting cascades process are always required a long time to accomplish the hemostasis. In other words, without the assistance of hemostatic devices and hemostatic agents, hemostasis cannot be timely conducted especially in emergency situations. Traditionally, sutures and staples are two main methods of achieving wound closure or implant fixation, whereas they can easily cause the additional trauma, leakage and lack of tissue integration due to the obviously inherent mismatch between body tissues and fixture compliances (Ghobril and Grinstaff, 2015).

Hemostatic agents, sealants, adhesives and their compositions shall meet a wide range of requirements. In 2008, safety, efficacy, usability, cost, and Food and Drug Administration (FDA) approval are required as five main necessities for use all over the world (Spotnitz and Burks, 2008, 2010), and other specific requirements like biodegradability, biocompatibility, mechanics, swelling ratio, stability, qualified water tightness, adjustable adhesion, and enhanced ability of healing process through the delivery of growth factors or active drugs are also be focused for actual usage. Accordingly, ideal hemostatic agents should simultaneously have abilities with rapid hemostasis, good biocompatibility, well-matched degradation, no adverse effects on wound healing and conducive to accelerating the healing process (Howe and Cherpelis, 2013; Qin et al., 2015; Dowling et al., 2016; Momeni and Filiaggi, 2016). Furthermore, the important issues of quality, manufacturing cost, stability, swelling rate, safety and adjustable mechanics should also be considered and addressed (Hsu et al., 2015).

Until now, a varied of polysaccharide-derived hemostatic materials, like fibrinogen, albumin, thrombin, gelatin, collagen, chitosan, cellulose, dextran alginate, starch, and hyaluronic acid, have been well-developed as local hemostatic agents, tissue adhesives and sealants in biomedical fields (Low et al., 1993; Liu, 2000; Milkes et al., 2002; Hung-Hsing and David, 2003; Oz et al., 2003; Sabel and Stummer, 2004; Liu et al., 2005; Lew and Weaver, 2008; Azargoon et al., 2011; Pozza and Millner, 2011; Rajagopal and Hakim, 2011; Fortelny et al., 2012; Vyas and Saha, 2013; Behrens et al., 2015; Zeng et al., 2016). While the synthesized hemostatic materials of isocyanate, polyethylene glycol and catechol monomers exhibit favorable adhesion and antibacterial properties for wide applications (Napoleone et al., 2009; Bochyńska et al., 2013; Boerman et al., 2017). In addition, some inorganic hemostatic materials including kaolin, mineral zeolite, montmorillonite, and bioactive glass are attributed to their high pore structure and fast water absorption (Gerlach et al., 2010; Ran et al., 2010; Arnaud et al., 2011; Pourshahrestani et al., 2016). So far, biodegradable self-assembling peptide hydrogels are another kind of hemostasis that possess a similar morphology to fibrin clots for capturing the blood components (Saini et al., 2016). Although there are a series of synthetic polymer and polysaccharide-based hemostatic materials on the market (Table 1), some important issues of biosafety, hemostatic effect and high cost still greatly limit their widespread biomedical applications. For instance, famous blood functional components of fibrinogen and thrombin, biologically derived hemostatic agents, have expensive costs, short-shelf life, and potential side-effects of virus contamination (DeAnglis et al., 2017). For the synthetic adhesives, some obvious disadvantage of non-biodegradability and potential cytotoxicity should be issued as applied in clinical use. For inorganic hemostatic materials, high exothermic reactivity and poor biodegradability can easily cause thermal damage and inflammatory reactions for the clinical patients.

**TABLE 1:** Commercially available synthetic polymer and polysaccharide-based tissue adhesives.

Commercial Product	Approved Indications	Constituents
FocalSeal-L (Focal Inc.), replaced AdvaSeal (Sawhney et al., 1993)	Sealing lung air leaks	Photopolymerizable PEG-co-poly (lactic acid)/poly(trimethy lene carbonate)
DuraSeal (Covidien), DuraSeal Xact (Preul et al., 2003; Cosgrove et al., 2007)	Adjunct to sutures for dural repair; anti-adhesion (SprayShield); retina reattachment; nerve sciatic anastomosis; vascular closure	Tetra-NHS- PEG and trilysine
CoSeal (Cohesion Technologies) (Wallace et al., 2001)	Adjunct hemostasis in vascular surgery; inhibiting suture line bleeding	Tetra-NHS-PEG and tetra-SH-PEG
SprayGel (Covidien) (Dunn et al., 2001b; Ferland et al., 2001; Johns et al., 2003)	Adhesion barrier in gynecological and colorectal procedures	Tetra-NHS-PEG and tetra-NH_2_-derivatized PEG
TissuGlu^®^ (Gilbert et al., 2008)	Prevention of seroma formation under skin flaps	Lysine di/tri isocyanate-PEG polymers
TissuePatch (TissueMed) (Kettlewell et al., 2007; von der Brelie et al., 2012; Ferroli et al., 2013)	Air leakage in thoracic surgery; sealing and reinforcing soft tissues adjunct to sutures; dural repair in cranial surgery, adjunct to sutures	Poly-((N-vinylpyrrolidone)_50_-co-(acrylic acid)_25_-co-(acrylic acid N-hydroxysuccin imide ester)_25_)
HemCon Bandage Pro (Jayakumar et al., 2011)	Hemostasis; antibacterial barrier	Chitosan
Commercially unavailable (Ono et al., 2000; Ishihara, 2002; Ishihara et al., 2002)	Sealing suture lines in aorta and intestine; incision sealing in trachea	2% of amines of chitosan substituted lactobionic acid, p-azido-benzoic acid
Actamax (Bhatia et al., 2007b)	Adhesion prevention; tissue sealing	Dextran aldehyde, 8-arm-NH_2_-PEG functionalized with tris(2-aminoethyl) amine
CS Glue (commercially unavailable) (Wang et al., 2007)	Connecting biomaterial to cartilage	Chondroitin sulfate functionalized with both aldehyde and acrylate groups

In the present review, we will describe the current polymeric adhesives and hemostatic sealant in surgical toolkits including the commercially available materials and recently reported adhesives in literatures for wound closure as well as their respective advantages and drawbacks. From a point of view of polymer chemistry, polymeric hemostatic materials will be divided into two categories: synthetic adhesive (polycyanoacrylates, poly(ethylene glycol), polyurethanes and polyesters) and polysaccharide (chitosan, cellulose, alginate, hyaluronic acid, starch, and chondroitin). Although we are intended to quantitatively compare these various biomaterials, unfortunately many reports are only provided with the qualitative results at various test conditions. Therefore, the purpose of this review is to highlight the scientific progress over the years and further summarize the surgical applications of most crucial adhesives and sealing biomaterials from the synthetic and polysaccharide adhesives, thereby proposing the importance, necessity and urgency of continuous research of advanced bio-adhesives for the biological hemostatic applications.

## Synthetic Polymers-Based Hemostatic Adhesives

### Polycyanoacrylates

Cyanoacrylate-derived tissue adhesives are a series of synthetic sealants with instantaneously strong adhesion force and rapid adhesive time, which are simply polymerized at room temperature without any harsh conditions of solvent, heating, light, catalyst, pressure, etc. In this process, cyanoacrylates can be easily generated within seconds by exposure to a small amount of water to initiate the polymerization and form strong bond interactions to yield polycyanoacrylate adhesives in a single-component system. The mechanism of the cyanoacrylate tissue adhesion is ascribed to the rapid formation of covalent bonds between the cyanoacrylate and amine groups within the tissue proteins (e.g., primary amine of lysine). As it is applied to tissues, cyanoacrylate monomer will penetration into cracks of tissue surface in order that a firm bond between the tissue and the glue is rapidly formed. In addition, mechanical interlocking force between the cyanoacrylate glue and tissues also plays vital roles in strength enhancement. Importantly, these cyanoacrylate adhesives are even useful on the moist and porous substrates (Tseng et al., 1990; Celik et al., 1991; Evans et al., 1999; Fortelny et al., 2007).

**FIGURE 1:**
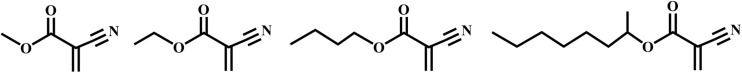
Cyanoacrylate monomers for tissue adhesives, including methyl-cyanoacrylate, ethyl-cyanoacrylate, butyl cyanoacrylate and octyl cyanoacrylate. Reproduced from Singer et al. (2008) with permission from Copyright 2008 Elsevier.

However, fast degradation of polycyanoacrylates via the hydrolysis effects generates a lot of toxic products (cyanoacetate and formaldehyde) that can result in severely inflammatory responses and wound infection for the cells and tissues (Trott, 1997). The degradation rate decreases with steric hindrance of long alkyl chains of cyanoacrylate polymers (Figure 1), and thus shorter chains of polycyanoacrylate derivatives can cause higher concentrations of toxic products for endanger the human health. Besides, polymercyanoacrylate adhesives possess weak mechanical strength, for example, the ethyl- and butyl-cyanoacrylates become brittle and fragile after the polymerization, which cannot be suitable for the use of long incisions or skin creases (Singer et al., 2008; Dragu et al., 2009).

Although some cyanoacrylate tissue adhesives are approved by FDA and commercially used for the closure of a local skin incision and barrier of a microbial penetration, they remain the inflammatory responses that may inhibit the collagen reconstruction and wound repair (Montanaro et al., 2001; Singer et al., 2008; Bhatia, 2010).

### Poly(ethylene glycol)

**FIGURE 2:**
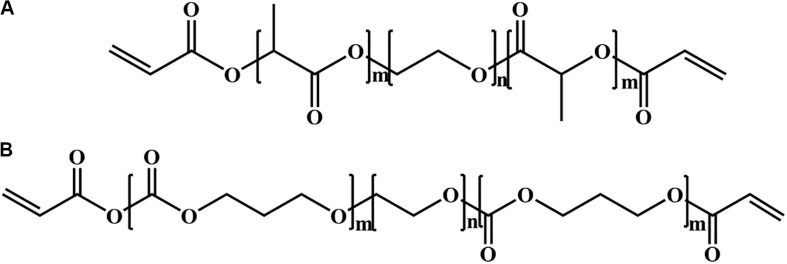
Two different building blocks of photopolymerizable copolymer for PEG photopolymerizable tissue adhesives with **(A)** PLA-PEG-PLA and **(B)** PTMC-PEG-PTMC diacrylates.

Poly(ethylene glycol) (PEG) is hydrophilic and biocompatible polymer with a stealth-like behavior *in vivo*, and can be employed as another important class of tissue adhesives (Knop et al., 2010; Wang et al., 2015, 2018; Cao et al., 2018; Bian et al., 2020; Li et al., 2020; Tang et al., 2020; Yu et al., 2020). There are three main kinds of PEG-based tissue adhesives, including the photopolymerizable adhesives (FocalSeal^®^, successor of AdvaSeal), PEG-trilysine adhesives (DuraSealTM) and functionalized PEG with two component adhesives (CoSeal^®^, SprayGel). The first PEG-based adhesive consists of an ABA-triblock polymer, wherein PEG block is middle and poly(lactic acid) (PLA) or poly(glycolic acid) (PGA) blocks are outer via ring opening polymerization of lactide or glycolide with a bifunctional PEG macroinitiator in Figure 2A, followed by the end-functionalized modification of photopolymerizable acrylate moieties. In aqueous solutions, these copolymers self-assemble into the micellar gels, enabling the fast photopolymerization due to the high density of acrylate concentrations within the hydrophobic area. Although this hydrogel is non-adhesive to tissues, it still exhibits strong adhesion even on the smooth surface, which ascribes to the creation of interpenetrating networks with the tissue proteins and generation of an adhered complex after the direct polymerization in contact to tissue.

Based on the self-assembly principle in aqueous solutions, FocalSeal^®^ is approved as a commercial sealant by the FDA in 2000 to seal air leaks after the lung surgery (Anonymous and Confluent Surgical Inc, 2004). To improve its mechanical properties, a second ABA triblock copolymer of poly(trimethylene carbonate) (PTMC)-PEG-PTMC is added (Figure 2B) to form the hydrogels by crosslinking acrylate groups with more than 80 wt% of water content after the photopolymerization (Macchiarini et al., 1999; Anonymous and Focal Inc, 2000; Quinn, 2005). After the hydrolysis of ester bonds and degradation of sealant hydrogels, the degraded products of LA and PEG are released, metabolized and cleared through the kidneys (Macchiarini et al., 1999). Furthermore, other PEG sealants are also reported according to the same principle, such as poly(propylene fumarate) (PPF)-PEG-PPF, poly(succinic acid) (PSA)-PEG-PSA, etc. (Suggs et al., 1998; Tanaka et al., 1999; Nivasu et al., 2004). However, on account of the irradiation condition for these PEG photopolymerizable tissue adhesives, they are not fully safe for usage *in vivo* and not widespread securely. Also, these produced free radicals in the polymerization may bring about unknown hazard and side reaction to the tissues.

**FIGURE 3:**
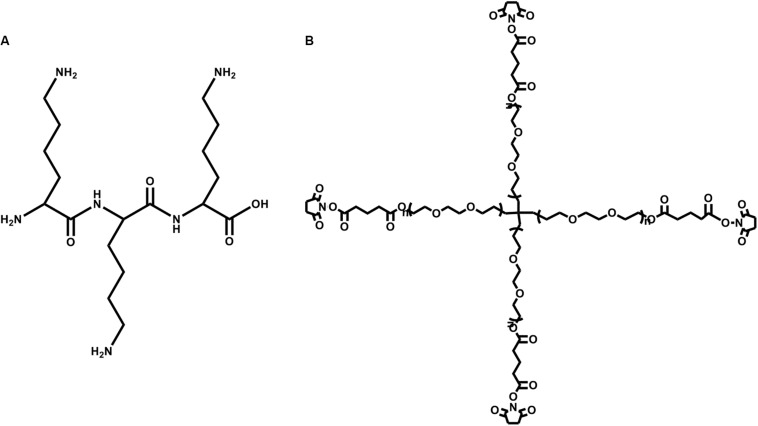
The structures of **(A)** trilysine and **(B)** pentaerythritol poly(ethylene glycol) ether succinimidyl glutarate.

**FIGURE 4:**
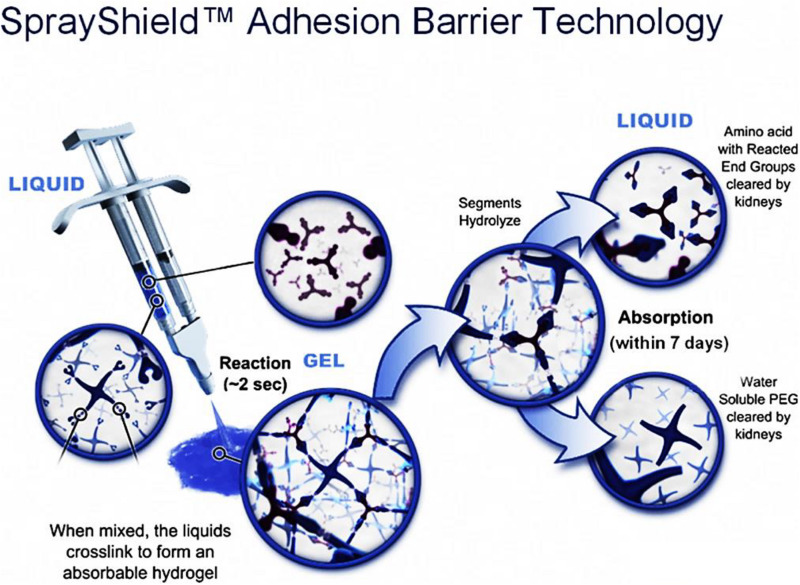
A schematic overview of DuraSealTM with a two-component system of tetra-PEG and trilysine. Reproduced from Ghobril and Grinstaff (2015) with permission from Copyright 2015 Royal Society of Chemistry.

The second PEG-based tissue adhesive, known as DuraSealTM Dural Sealant device, is first used to prevent cerebrospinal fluid (CSF) leakage for the cranial surgery (Cosgrove et al., 2007). DuraSeal is formed as two varied components are touched, which is composed of a tetra-amine crosslinker of trilysine at a dissolved sodium borate buffer (pH 10.2) and a tetra-armed PEG (M_*n*_ = 10 kDa) capped with N-hydroxy succinimide-esters (NHS) in a sodium phosphate buffer (pH 4.0) in Figure 3. As two components are simultaneously sprayed to the tissues, amine groups of trilysine are quickly reacted with NHS groups to generate amide bonds crosslinked network (Figure 4). Notably, upon application of this DuraSeal adhesive to the tissues, the amine and thiol groups of proteins in tissue surface can also simultaneously react with NHS-functionalized polymer, generating the strong covalent adhesion to the tissues (Boogaarts et al., 2005).

Similarly, along with the ester hydrolysis and the enzymatic degradation of lysine ingredients, DuraSealTM can degrade after 4–8 weeks with the removal of degradation byproducts through the renal clearance from the body (Ghobril and Grinstaff, 2015). However, the drawback of these tissue adhesives is high swelling ratio that may hinder the use in bone regeneration because of the potential oppression to the nerves (Lee et al., 2010). A modified two compound adhesive DuraSealTM of Xact Adhesion Barrier and Sealant System can decrease the swelling behaviors by inserting more crosslinkers to change ratio of PEG versus trilysine and obtain a higher crosslinking degrees (Anonymous and Confluent Surgical Inc, 2009). A inconvenient downside of DuraSeal is the required two-component design that can easily cause the syringe clogging in the mixed component process if these tissue adhesives are not applied immediately enough.

**FIGURE 5:**
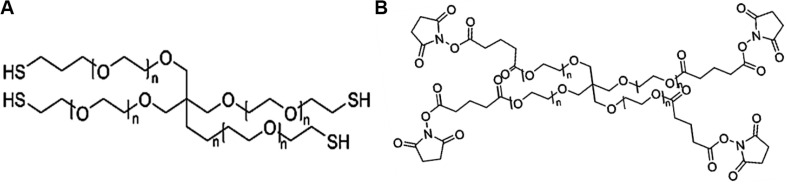
The chemical structures of **(A)** pentaerythritol poly(ethylene glycol) ether tetrathiol and **(B)** pentaerythritol poly(ethylene glycol) ether tetrasuccinimidyl glutarate.

The third PEG-based adhesive is another two-component tissue sealant of PEG-PEG adhesives, analogous to CoSeal^®^ Surgical Sealant. It is composed of a 20% (w/v) solution of a tetra-PEG-SH in a pH 9.6 of sodium phosphate/sodium carbonate buffer and a second 20% (w/v) solution of a tetra-PEG-NHS in pH 6.0 of sodium phosphate buffer in Figure 5 (Goode et al., 2001). Once mixing these two PEG solutions, thiol can react with NHS groups to generate a thioester bond and form a well-organized crosslinked network within 3 s along with the simultaneous formation of a small number of disulfide linkages. During this process, a transamidation reaction occurs between amines and thioesters to form the covalent bonds in the tetra-PEG network between the adhesives and tissues (Wallace et al., 2001). Noted that even though the CoSeal^®^ adhesive is applied onto the non-reactive surfaces, the produced adhesions are still highly stiff because the permeation of liquids flow into the crack and fracture of materials. The degradation time of this hydrogel was within several weeks due to the hydrolysis of glutarate esters and thioesters (Goode et al., 2001). Compared to the DuraSeal with amide bonds, faster degradation ascribes to the unstable thioester groups. However, this PEG-PEG hydrogel still possesses high swelling ratio and relatively weak adhesion to the surrounding tissues (Saunders et al., 2009). SprayGel adhesion barrier system is another developed example of PEG-PEG sealants, which is also composed of two reactive tetra-PEG-NHS and tetra-PEG-NH_2_ solutions. Once mixing two solutions together, the adhesives are also quickly formed and degraded after 5–7 days with the excretion to outside of body by the renal clearance (Dunn et al., 2001a; Ferland et al., 2001; Johns et al., 2003).

### Polyurethanes

These synthetic polyurethanes are widely applied for various adhesives due to the excellent thermal stability in the physiological temperature and the absence of hemolysis (Ferreira et al., 2007), wherein TissuGlu^®^ is a popularly surgical adhesive to bind abdominal tissues. However, a common side effect of abdominal surgery is a subcutaneous effusion under the skin to cause the seroma, which requires to drain regularly to clear fluids. In general, the abdominal skin must be reattached to the underlying layer during the abdominal surgery, but meanwhile an imperfect connection may cause the gaps between the subcutaneous tissues and the skins. Postoperative effusion may ascribe to the presence of this cavity. As a result, TissuGlu^®^ adhesive are widely used to shorten the space cavities via the formation of a bond among these tissue layers (Gilbert et al., 2008).

TissuGlu^®^ surgical adhesive consists of a hyperbranched macromolecules with isocyanate groups and ca. 50 wt% of lysine, and the polyurethane prepolymer can be generated via an organized combination of lysine diisocyanate and triisocyanate with diols and polyols (Beckman, 2011). When this prepolymer is touched with water within the tissue, it hydrolyzes into an amine through the isocyanate group and reacts with the surplus isocyanates to construct the crosslinking networks through the urethane bonds, which requires as long as 25 min to provide enough surgeons time to close the abdominal skin. On account of the hydrolysis effect and enzymatic degradation of lysine-based bonds, this surgical adhesive can give rise to many byproducts of glycerol, lysine, ethanol, and carbon dioxide with the ready clearness from the body. Trials on human patients have shown that TissuGlu^®^ adhesive is biocompatible enough to achieve the reduction of fluid accumulation extents (Walgenbach et al., 2012; Ohlinger et al., 2018). Afterward, a new single component of Sylys^®^ surgical sealant is developed using the urethane chemistry by Cohera Medical Inc., which can provide supports after anastomosis to prevent the leakage.

**FIGURE 6:**
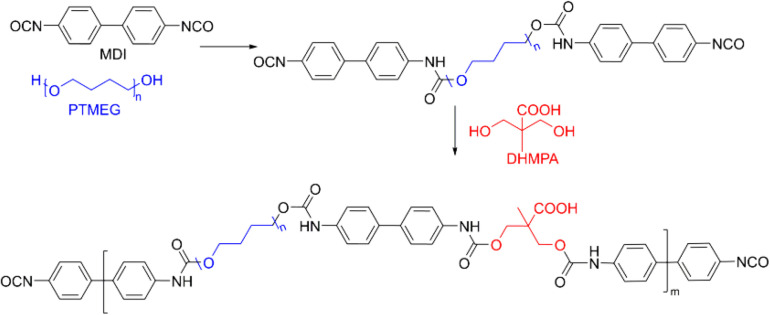
A typical polyurethane with the main components of MDI (black), PTMEG (blue), and DHMPA (red). Reproduced from Phaneuf et al. (2001) with permission from Copyright 2001 Elsevier.

Polyurethanes can also be widely applied for the wound hemostasis, bone fixation and vascular graft sealants (Lipatova, 1986; Phaneuf et al., 2001; Ferreira et al., 2008b). Since vascular graft can slightly permeate into the blood and induce the leak to the whole body, these polyurethane sealants are required to tight the water for actual applications. For example, a polyurethane product has been prepared by the reaction of 4,4-diphenylmethane diisocyanate (MDI) and poly(tetramethylene ether glycol) (PTMEG) followed by adding the 2,2-bis(hydroxymethyl)-propionic acid (DHMPA) in Figure 6 (Phaneuf et al., 2001). Besides, the sealant should also be bound with proteins within the bloodstream to provide the effective blood-biomaterial interactions. To date, no vivo papers are yet demonstrated, which may ascribe to the accumulation problem of hydrophobic and stable character of PTMEG *in vivo*.

### Polyesters

Aliphatic polyesters like polycaprolactone (PCL) and polylactic acid-glycolic acid (PLGA) are significantly applied as tissue adhesives for various biomedical applications. Ferreira et al. (2008b) functionalized PCL with isophorone diisocyanate (IPD) and hexamethyldiisocyanate (HDI) to obtain several tissue reactive polymers. After placing them between two gelatin pieces and separating gelatin sheets, the adhesive tests found that the IPD-modified PCL can effectively bind to the gelatin parts without affecting the adhesive sections. However, HDI-modified PCL exhibited failure adhesive properties because of lower NCO concentration within polymers (Ferreira et al., 2008b). Besides for the dependence on the chain entanglements of linear chains, the absence of crosslinks was the main reason for the potential limitation. Although some adhesive strength of PCL-based materials had been developed by means of strong interpenetration between the polymeric crosslinking networks and the tissues, few literatures of *in vivo* tests are reported so far (Ferreira et al., 2008a).

Two examples of PLGA-based adhesives are so-called TissuePatchTM for the prevention of air leakage after lung surgery and TissuePatchDuralTM for the prevention of fluid leakage after brain surgery (von der Brelie et al., 2012; Ferroli et al., 2013). These adhesive patches are composed of poly((N-vinylpyrrolidone)_50_-(acrylic acid)_25_-(acrylicacid NHS-ester)_25_) and PLGA with multiple layers. TissuePatchTM contains four layers, of which the second and third layers are NHS-functional polymers, and the first and fourth layers are PLGA, interspersed with NHS functional polymers (Kettlewell et al., 2007). As this adhesive patch is attached to the tissue proteins, it can be reacted with the amine to form an amide bond between the patch and tissue within a minute. The adhesive tape can degrade after the hydrolysis of amide bond and PLGA *in vivo* for 50 days (Della Puppa et al., 2010). The main advantage is ease of use without tedious preparation before the operation.

## Polysaccharide-Based Hemostatic Adhesives

Polysaccharides are a kind of naturally derived polymers with sugar building blocks, which possess more exceptional advantages on the rich source of naturally raw materials, biodegradability, biosafety, good biocompatibility, no immune response or histologic reaction *in vivo*, etc. More importantly, these polysaccharide-based biomaterials can be feasibly synthesized and modified through simple physical and chemical methods for the hemostatic applications (Basu et al., 2015). Early in 1940s, Frantz (1948) prepared a locally absorbable hemostatic agent by oxidizing the cellulose, and then developed hemostatic alginate agents. Afterward, with the development of science and technology in the clinic field, polysaccharide-based biomaterials have produced a series of hemostatic agents tissue adhesives and sealants with good biosafety and biodegradability *in vivo* (Lewis et al., 2015). In this section, we will investigate and discuss some typical polysaccharide-based materials like chitosan, cellulose, alginate, hyaluronic acid and starch for the hemostatic applications (Table 2).

**TABLE 2:** Polysaccharide-based hemostatic adhesives.

Main component	Active ingredients	Clotting mechanism
Chitosan (Malette et al., 1983)	Positive amino groups	Adsorb positively charged platelets and red blood cells
Cellulose (Cheng et al., 2013)	Carboxyl groups	Binding iron ions in hemoglobin, activation of clotting factor VIII and promote platelet adhesion
Dextran (Bouten et al., 2014)	Hydroxyl in the ortho	Provide polyaldehyde seats
Alginate (Hama et al., 2010)	Linear polysaccharides	Rapid glue formation with tissue adhesion
Starch (Antisdel et al., 2009)	Polydextrose with numerous hydroxyl groups	Rapid water absorption and platelet coagulation
Hyaluronic acid (An et al., 2019)	Acid mucopolysaccharide	Carry a lot of water

### Chitosan

Chitosan (CS), a positively charged polysaccharide from chitin deacetylation, has greatly applied values in the biomedical fields owing to its good biodegradability, non-toxicity, antibiosis and non-antigenicity (Liu et al., 2020). In the 1980s, Malette et al. (1983) used the chitosan powders to apply for the hemostasis of open wounds due to the electrostatic interaction with the erythrocytes and manual compression to accelerate blood clotting (Brandenberg et al., 1984). FDA has approved two hemostatic agents of CloSur PAD and Hemcon chitosan, which can stop blood loss via the platelet aggregation effects (Lan et al., 2015; Kavitha Sankar et al., 2017). It is mentioned that varied degrees of deacetylation (DDA) and molecular weights (Mw) of chitosan display distinct hemostatic properties, so a mixed component of chitosan with variational DDA (75–88%) and Mw (8.6–247 kDa) is generally required (Hattori and Ishihara, 2015). Although chitosan hemostasis can accelerate erythrocyte adhesion and platelet activation, they also restrain the activation of contact system that is related to the intrinsic coagulation cascade and eventual thrombin formation, just like a double-edged sword in the hemostatic application (He et al., 2013).

**FIGURE 7:**
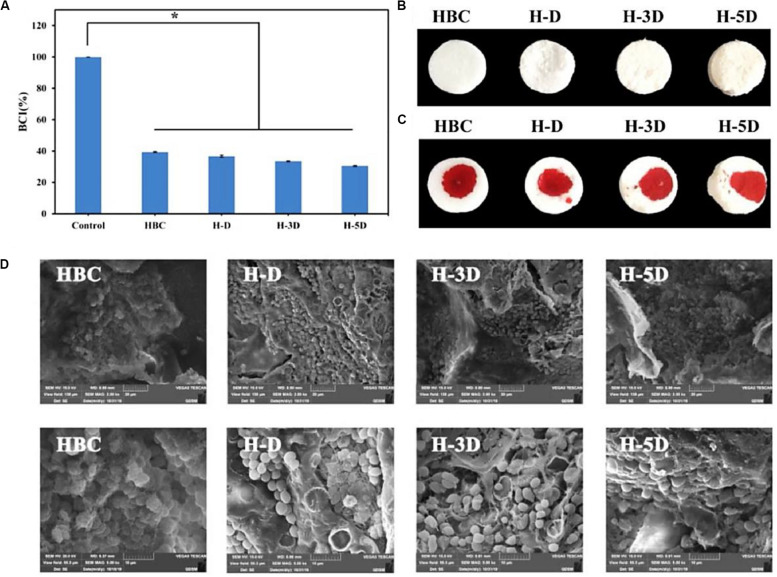
**(A)** BCI ration of HBC, H-D, H-3D, and H-5D. The data represent mean ± SD (*n* = 3), *p* < 0.05. The photo of composite sponges **(B)** without and **(C)** with blood. **(D)** SEM of red blood cell aggregation on HBC and H-Ds. Reproduced from Zhang et al. (2020) with permission from Copyright 2020 Elsevier.

On account of the high reactive amine groups within the chitosan backbone, CS is easily modified for improving the hemostatic efficacy (Yang et al., 2018). Dowling et al. (2016) synthesized the dodecyl-modified chitosan (HM-CS) via reacting with the amino groups. Dodecyl-modified chitosan was made into the self-expanding foam with a sprayed behavior from a gas tank. When the injured area was incompressible like the internal injuries in the trunk, this product could treat bleeding. Once spraying the foam into the open cavity, it could quickly form a barrier to prevent blood out of the cavity, which relied on the physical wrap of blood components into the formation of clusters via the hydrophobic interactions. Notably, this hemostasis foam could stop bleeding quickly without additionally external pressure. Yin et al. (2020) synthesized a nanofibrous polyvinyl alcohol (PVA)/quaternary ammonium N-halamine chitosan (CSENDMH) membrane for the hemostasis dressing. This membrane with a bead-free network and porous structure exhibited a good water absorption and excellent blood clotting abilities for the effective hemostatic applications (Yin et al., 2020). Zhang et al. (2020) designed a composite sponge of hydroxybutyl chitosan (HBC) and diatom-biosilica (DB) to improve the hemostatic effects (Figure 7). By means of its porous structures, good biocompatibility and fast fluid absorbability, H-D exhibited effective hemostasis effect with a shorten clotting time of 70% compared to the controls, because strong interface effect from H-D could induce the red blood cell absorption, active the inherent blood clotting pathway and accelerate the blood coagulation (Zhang et al., 2020). Liu et al. (2014) prepared a porous chitosan sponge by introduction of the halloysite nanotubes, which could significantly improve the clotting efficiency and promote the would repair than the pure CS. Kumar et al. (2012) reported a composite bandage with the main components of porous CS hydrogel/zinc oxide nanoparticles, which could increase swelling property, blood clotting rate and antibacterial ability.

### Cellulose

Cellulose, a main component of plant cell wall, is a kind of D-glucopyranose homopolysaccharide. In particular, cellulose oxide (OC), also known as cellulose oxide, is a denatured polysaccharide by the chemical modification of cellulose. Cellulose and its derivatives are popularly utilized as absorbable wound dressings and hemostatic products due to their excellent biocompatibility, biodegradability and low costs (Cheng et al., 2013; Metaxa et al., 2014; Kwak et al., 2015; Mertaniemi et al., 2016). Cellulose oxide can quickly absorb the liquids, entrap the platelets and erythrocyte, increase the concentration of clotting factors and speed up the clotting process as it is employed at the bleeding sites, facilitating the fibrin clots and blocking blood flow effectively (Hutchinson et al., 2013). Meanwhile, their carboxyl groups can initiate the coagulation by self-activation of coagulation factor XII.

Although OC had been extensively investigated as a hemostatic agent, it possessed obvious clinical disadvantages originating from the low pH of many carboxyl groups, which significantly limited the sensitive tissue (nervous and cardiac systems) therapy (Ohta et al., 2015). To address this problem, scientists have spent efforts on the improvement of its hemostatic applications. Demirekin et al. (2015) reported a potassium and sodium salt of ORC in the presence of metal ions to effectively accelerate the blood coagulation and inhibit bacterial infection. In addition, introduction of other polysaccharide is an effective method to enhance the hemostatic therapy. For example, He et al. (2014) reported a hemostatic agent by blending the chitosan on the surface of ORC gauze, exhibiting satisfactory hemostatic effect compared to the traditional ORC gauze. In addition, Karahaliloglu et al. (2017) fabricated a bilayer of wound dressing with CS and bacterial cellulose blends in the sublayer of and silk fibroin (SF) in the upper layer. When it was applied in the wound, the sublayer bacterial cellulose can quickly absorb a lot of liquid in the blood and the upper layer of SF can rapidly cause the platelet adhesion, which ascribed to the similar hierarchical structure to collagen/elastin fibers with high surface area/volume areas. Compared with the control of standard gauze, this bilayer dressing presented the highly effective hemostatic effect both *in vitro* and *in vivo* (Karahaliloglu et al., 2017).

### Dextran

**FIGURE 8:**
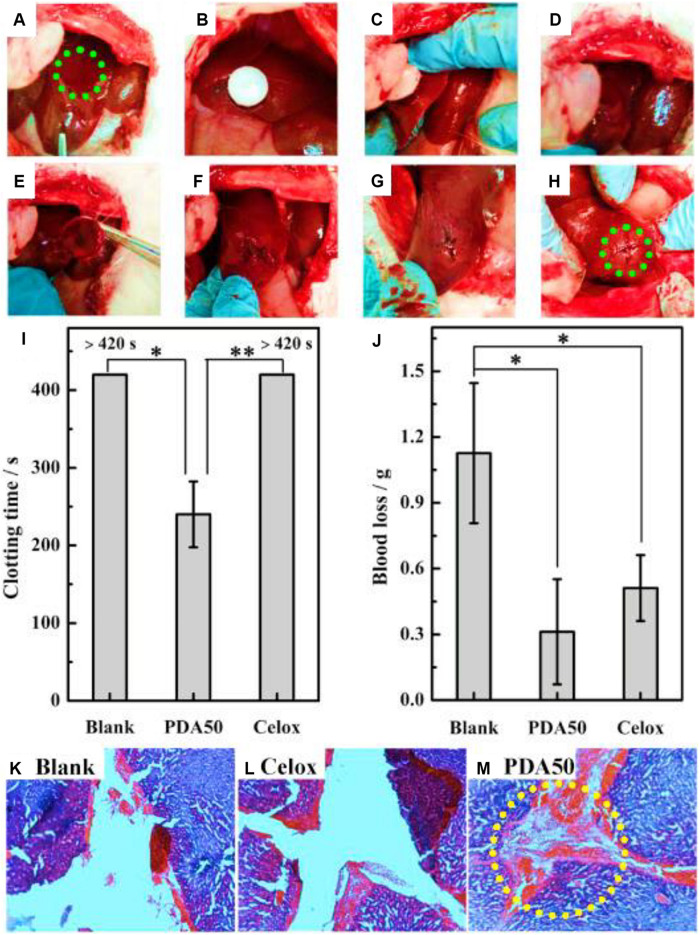
Hemostasis of liver injury using the rabbit model. **(A)** Creation of liver injury in the left medial lobe. **(B–D)** Treatment with PDA sponge. **(E–G)** Hemostasis maintained after removal of sponge. **(H)** Hemostasis kept even squeezing the wound. **(I,J)** Coagulation time and blood loss of liver injury. **(K–M)** Histopathology of liver trauma. Reproduced from Liu et al. (2019) with permission from Copyright 2019 Elsevier.

Dextran, a biocompatible polysaccharide, is composed of an α-1,6-linked D-glucopyranose residue. Like other polysaccharides, dextran has a large number of hydroxyl groups in its anhydroglucose unit with facile chemical modification. In addition, its high water absorption endowed the dextran with hemostatic function as a tissue adhesive agent (Bouten et al., 2014; Yan et al., 2017). Generally, NaIO_4_ is used to oxidize the adjacent diols of dextran into aldehyde groups, which can be chemically crosslinked with the amino groups of biomaterials or tissue proteins, exhibiting strong adhesive force for tissue sealants. When the dextran is oxidized less than 60%, it can slowly bind into the tissues, because the tissue-material adhesion force, local inflammation and systemic tissue toxicity is extensively relied on the number and density of aldehyde groups (Bhatia et al., 2007a). Liu et al. (2019) designed a kind of aldehyde dextran (PDA) sponge with good water absorption and adhesive behaviors (Figure 8). After optimization of pore size, PDA sponge displayed the quick blood absorption, powerful tissue adhesion and effective hemostasis on the rabbit models, because the quick coagulation process of PDA sponge could accelerate the wound block, cell aggregation and cell initiation without the need for coagulation cascade activation (Liu et al., 2019).

Artzi et al. (2009, 2011) prepared a kind of sealant consisting of star-shaped PEG-NH_2_ and Dex-CHO with various molecular weights and aldehyde oxidation degrees, exhibiting effective tissue adhesion behaviors after chemical modification. Du et al. (2019) reported a novel hydrogel dressing comprising hydrophobicity-modified CS and oxidized dextran. After the analysis of gelation behavior, self repair and rheological property, this hydrogel dressing presented good hemostatic and antibacterial activity in a rat hemorrhaging liver model, which demonstrated its multifunctional activities on the improvement of hemorrhagic and infected wound therapy (Du et al., 2019).

Although sealants and tissue adhesives based on oxidized dextran have been extensively investigated, the formation of imine bonds is an equilibrium reaction with instability in aqueous solutions. Wang et al. (2012) have developed a tissue glue consisting of the aldehyde dextran and gelatin. Incorporation of 2-isocyanoethyl methacrylate into the architectural backbone of dextran hydrogel can significantly increase the crosslinking degree along with formation of a dense intermolecular network, thus improving the mechanical strength and stability of biocompatible hydrogels (Wang et al., 2012).

### Alginate

Alginate consists of α-L-glucuronic acid and β-D-mannuroic acid monomers. On account of its good biocompatibility and biodegradability, alginate is easily formed to an ionic hydrogel or a microsphere crosslinked by Ca^2+^ ions (e.g., calcium alginate) for biomedical applications (Hama et al., 2010; Kinaci et al., 2013; Pinkas and Zilberman, 2014; van Elk et al., 2015). Once calcium alginate comes to contact with blood, Ca^2+^ ions can release in exchange for sodium ions, which can simultaneously accelerate platelet aggregation to activate the coagulation process and serve as a cofactor in the coagulation cascade. In addition, by means of the high water absorption, the modified CA can quickly attach the materials onto wound with a suitable hemostatic property. Shi et al. (2016) designed a kind of biodegradable and hemostatic composite microspheres comprising carboxymethyl chitosan, sodium alginate and collagen, which possessed high-efficient hemostatic property via the feasible platelet adherence, aggregation and activation.

**FIGURE 9:**
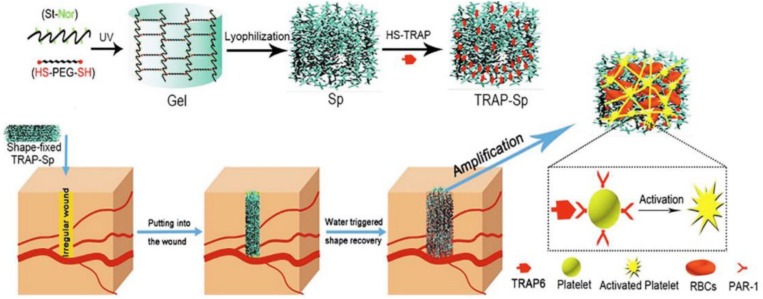
Preparation of TRAP-Sp with macroporous structures and reasonable mechanical properties. Reproduced with permission. Reproduced from Yang et al. (2019) with permission from Copyright 2019 Elsevier.

The drug loading capacity of alginate microspheres has also attracted people’s attention. Rong et al. (2015) prepared a thrombin-loaded alginate calcium microsphere via the emulsion crosslinking technique, which can transport the hemostatic agent for the blunt injury and abdominal solid viscera bleeding. Zhai et al. (2019) demonstrated a co-assembly system of peptide binding compound and alginatewith attractive cell adhesions, which exhibited the effective hemostatic property without adding other growth factors (Figure 9). This composite hydrogel can quickly stop bleeding after adding whole blood *in vitro*, and reduce the bleeding volume of the mouse liver puncture model to about 18% of the untreated group. Meanwhile, it promoted the migration of fibroblasts and accelerates wound healing speed of the mouse full-thickness skin defect model, which was developed into the promising nanocomposite materials for a variety of biomedical applications (Zhai et al., 2019). Huang et al. (2019) prepared a hemostatic composite (SACC) microsphere via the crosslinked technology of sodium alginate (SA), carboxymethyl chitosan (CMC) and collagen. On account of generic and narrow sphere shape, rough surface and high-water absorption, SACC showed better hemostatic effects than that of CMC and SA using the bleeding rat models. In addition, SACC exhibited good biocompatibility and biodegradability by histomorphological and immunofluorescent results, which can be used in the future clinical hemostasis applications (Huang et al., 2019).

### Starch

Starch is a widely natural polymer with high water solubility and low cost. Starch can be modified into the gelatinized starch, grafted starch and crosslinked starch by means of simply chemical methods, and degraded into oligosaccharides, maltose and glucose by plasma amylase *in vivo*. In recent years, starch microspheres (DSMs) are widely applied in the temporary blockage of blood vessels in combination with cytotoxic drugs in the treatment of malignant tumors. HemoStase and Arista are two commercially available starch-based hemostatic agents (Humphreys et al., 2008; Antisdel et al., 2009). To overcome its insufficient drawback for the severe bleeding, DSMs can be used in combination with recombinant factor VIIa, fibrinogen or thrombin to improve its hemostatic effect (Bjorses and Holst, 2007). However, these derivative agents may improve viral infections in clinical use. For example, Bjorses et al. (2011) tailored surface properties (negative/positive charge and hydrophilic/hydrophobic ratio) of DSMs to affect the material/blood interactions, showing superior hemostatic capacity *in vivo*.

In addition, incompressible bleed still faces great challenges for irregular wound treatments. Yang et al. (2019) synthesized a hemostatic starch/PEG hemostatic sponge (TRAP-Sp) with good water absorption, passive hemostatic performance and rapid self-healing property to absorb the plasma, concentrate blood cells and improve the blood coagulation (Figure 9). Once applied and contacted with blood, this hemostatic sponge could quickly expand pressure onto the injured sites with outstanding mechanics and superior resilience (Yang et al., 2019).

### Hyaluronic Acid

**FIGURE 10:**
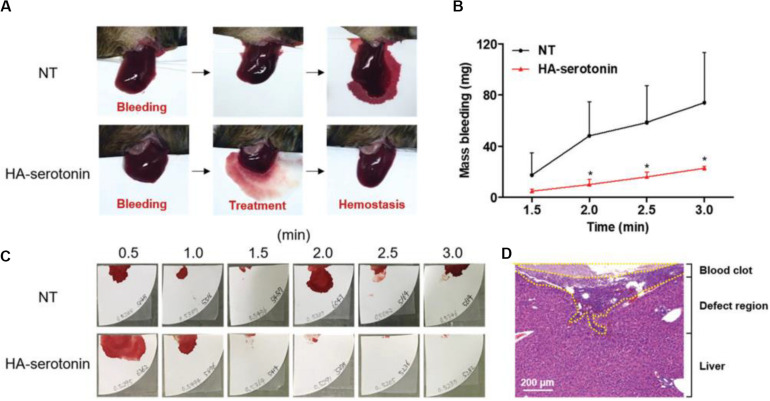
**(A)** HA-serotonin-mediated hemostasis in a liver hemorrhage model of Factor VIII-deficient hemophilia mice without treatment (NT, top) and treatment by hemostatic adhesives (HA-serotonin, bottom). **(B)** Accumulated blood loss from hemophilia mice after bleeding without any treatment (NT, black) and treatment of hemostatic adhesives (HA-serotonin, red) (*n* = 3, *p < 0.05 versus NT group). **(C)** Photographs of the blood absorbed papers from non- and HA-serotonin-treated hemophilia mice every 30 s up to 3 min. **(D)** H&E staining of damaged liver harvested from HA-serotonin-treated hemophilia mice for 3 days. Scale bar = 200 mm. Reproduced from An et al. (2019) with permission from Copyright 2019 Royal Society of Chemistry.

Hyaluronic acid (HA) consisting of D-glucuronic acid and N-acetyl-D-glucosamine is a linear non-sulfated polysaccharide, which can facilitate cell adhesion and migration because of excellent water retention and inherent swelling property *in vivo*, which contribute to the suitable conditions for wound repair and accelerate the collagen secretion from wound surface via the fibroblast proliferation effect. An et al. (2019) developed a new class of hemostatic adhesive using serotonin-conjugated HA hydrogel system, wherein the serotonin could promote the hemostasis of blood clotting in platelets. Inspired by platelet clotting mechanism, the serotonin-conjugated HA hydrogel showed superior hemostatic ability in normal and hemophilic lesions than the commercially fibrinolytic agents, which could prevent the abnormal post-hemostatic tissue adhesion in a rat model (Figure 10; An et al., 2019).

Luo et al. (2019) prepared two kind of injectable hydrogels of self-crosslinking gelatin and hyaluronic acid/gelatin for the hemorrhage control, which possessed good stability, low cytotoxicity, favorable bursting strength and excellent hemostatic ability compared to commercial fibrin glue (Luo et al., 2019). Hong et al. (2019) had developed a strongly adhesive hemostatic hydrogel for repairing arterial and cardiac hemorrhages. After ultraviolet irradiation of methyacrylated HA, it could quickly form the hydrogel, adhere and seal the bleeding arteries and heart walls. These repairs could withstand higher blood pressures than those of most traditionally clinical settings. Notably, hydrogel could prevent the hypertensive bleed from a 4–5 mm of incision wound in a pig carotid artery and hypertensive bleeding from a 6 mm of heart penetrating hole in a pig heart, presenting greatly clinical advantages for the wound sealants.

## Future Outlook and Conclusion

In this review, synthetic and polysaccharide adhesives have shown outstanding performance and multifunctionality when compared to the commercially available hemostatic polymers, but many challenges remain unresolved. One of the key issues is that the existed adhesives are lack of sufficient adhesion strength to replace the sutures, especially for the fragile tissues that need to close the leakage of liquids or gases. For synthetic polymers-based hemostatic adhesives, although the cyanoacrylate-based adhesive exhibited the incomparable hemostatic property than any other hemostatic agents, the biocompatibility profile of this cyanoacrylate did not meet the standardized guidelines to speed up regulatory approval process. Poly(ethylene glycol)-based hemostatic hydrogels have been used as a biodegradable adhesive to possess more advantages of being free of any human/animal materials, being safe and well-tolerated, and having a tight covalent bond and adhesion force to the surface of wet tissue. However, they often significantly swell *in vivo* and have undesirable mechanical strength for their applicability. In addition, PEG-based hydrogels are needed to produce *in situ* with two ingredients, which are relatively difficult to store separately as freeze-drying products and handle for usage. On account of the fast crosslinking of PEG hydrogels, these two ingredients are needed to first dissolve and then mix together through a dual syringe spray with a short handling time. Besides, these PEG hydrogel adhesives are quite expensive for limitation of wide use in the clinic applications. Therefore, great challenges about the synthetic adhesives is to design and prepare multifunctional polymers that simultaneous possessed safe, high strength and strong adhesion onto the tissues.

Polysaccharide-based adhesives have more advantages of intrinsic biocompatibility, safety and biodegradability, but they are always required to be modified to improve the solubility (e.g., chitin and chitosan) and further crosslinked with other polysaccharides. The huge benefits of polysaccharide-based adhesives are their widely biomedical fields from the former closing dura and corneal incisions to the current cartilage injures of glues and hemostatic products in regenerative medicine. As for the future research of polysaccharide-based hemostatic materials, pursuits of multifunctionality, and more advanced technologies are vital factors that should be issued. On the one hand, proven physic-chemical modification methods can furnish the polysaccharide with powerful adhesions with the tissue surfaces to acquire the directly rapid hemostasis without fully relying on the activation of coagulation process itself and inducement of inherent systemic emboli and thromboses. Furthermore, intelligent polysaccharide-based hemostatic materials should also facilitate the whole sequential processes of hemostatic, analgesia, anti-infection, inflammation, proliferation, remodeling, and healing functions to promote the long-term care of wound until body recovery. On the other hand, more advance techniques are urgently needed to endow the polysaccharide-based hemostatic materials with facile usages and great potentials. For example, by means of layer-by-layer self-assembly, electrostatic spinning, and reverse emulsion polymerization technologies, more inorganic nanomaterials can be blended into the biocompatible polysaccharide to well-organized into inorganic-organic hybrid biomaterials to directly and high-effectively activate the coagulation cascade and improve the hemostatic performance.

Future biomedical adhesives should be required with environmental stimulus responsiveness, which can respond to changes in the externally applied stimuli, such as pH, light, electricity, temperature and magnetism, or other active biomolecules (glucose, enzyme, etc.) within their surroundings. In this case, these changes can tailor the bioadhesives to release the encapsulated drug particles and improve the adhesive properties. Besides, scientists should also do consult the end-users about the feasibility and practicality of hemostatic materials in designing and fabricating novel adhesives. Researchers and clinicians need to work more closely together to develop high-level biological adhesives, identify unmet requirements and prioritize their design for further clinical applications on the market.

## Author Contributions

XW and JZ initiated the project. DL, JC, MZ, and CL searched the database, wrote, and finalized the manuscript. XW and JZ made suggestions and revised the article. All authors reviewed and commented on the entire manuscript.

## Conflict of Interest

The authors declare that the research was conducted in the absence of any commercial or financial relationships that could be construed as a potential conflict of interest.
